# Protein-coding genes in humans and model mammals (mouse, rat and pig): gene identifiers and disambiguation of gene nomenclature retrieved from the Ensembl genome browser

**DOI:** 10.1186/s12864-025-12329-8

**Published:** 2025-12-17

**Authors:** Grzegorz R. Juszczak, Chandra S. Pareek, Urszula Czarnik, Mariusz Pierzchała

**Affiliations:** 1 Department of Animal Behavior and Welfare, Institute of Genetics and Animal Biotechnology, Polish Academy of Sciences, Jastrzebiec, Poland; 2 Department of Infectious, Invasive Diseases and Veterinary Administration, Institute of Veterinary Medicine and Division of Functional Genomics in Biological and Biomedical Research, Centre for Modern Interdisciplinary Technologies, Nicolaus Copernicus University, Torun, 87-100 Poland; 3 Department of Pig Breeding, Department of Animal Biochemistry and Biotechnology, Faculty of Animal Bioengineering, University of Warmia and Mazury in Olsztyn, Ul. M. Oczapowskiego 5 Str, Olsztyn, 10-719 Poland; 4 Department of Genomics and Biodiversity, Institute of Genetics and Animal Biotechnology, Polish Academy of Sciences, Jastrzebiec, Poland

**Keywords:** Gene symbols, Ensembl, NCBI, RGD, HGNC, VGNC, Mouse, Rat, Pig, Human

## Abstract

Gene nomenclature contains current official symbols and various numbers of synonyms, which pose a challenge to integrating genomic data and increase the probability that different genes share the same symbol. Therefore, we retrieved identifiers assigned to all protein-coding genes in human, mouse, rat and pig genomes that are available in the Ensembl genome browser (release 113) to assess the number of genes, compare species and identify ambiguous symbols. Results: Our analysis revealed that the total number of symbols, both official symbols and synonyms, used to identify protein-coding genes ranges from 16,600 in pigs to 64,580 in mice. Furthermore, the gene nomenclature is not complete because there are also genes without an assigned symbol, which indicates gaps in understanding protein-coding genes, especially in pigs. We also found a large number of gene symbols that map to more than one gene. These symbols might complicate the identification of about 10% of rat and mouse genes and 18% of human protein-coding genes. A simple solution for this problem is the usage of stable gene IDs assigned by scientific institutions and committees (Ensembl, NCBI, RGD, HGNC and VGNC) provided that the genomic information associated with these IDs is retrieved directly from proprietary databases containing the most accurate data. Finally, although gene symbols may pose a problem with unequivocal identification of genes, there are instances when no other identifiers are available in the literature. Therefore, we have developed an R script performing search of the Ensembl database and integrating data to provide a single list of updated symbols with annotation about their ambiguity. Conclusions: Gene symbols are not always reliable and should be reported together with stable IDs to enable unequivocal identification of genes. Therefore, data containing only gene symbols should be used cautiously to avoid misidentification of genes. A solution for this problem is our R script REgeness that performs a gene symbol update to current official versions combined with identification of ambiguous symbols and retrieval of other IDs from the Ensembl database.

**Supplementary Information:**

The online version contains supplementary material available at 10.1186/s12864-025-12329-8.

## Background

A reliable presentation of results that prevents misunderstanding of published data is a core issue in science. Therefore, a considerable attention has been attracted by the problem of errors in gene symbols introduced by Excel [1–4]. However, another problem that is much less recognized is the usage of ambiguous gene symbols derived from abbreviated full gene names. Gene symbols are convenient and more reliable than full gene names [5] and, therefore, are commonly used to identify genes. However, for many genes there are multiple symbols, including current official symbols and synonyms that were used in the past, and, therefore, are present in the literature data. This state of affairs results from the history of gene discovery made by different research groups in different species, subsequent gradual refinements of gene names forced by a better understanding of their biological functions and, finally, standardization efforts. As a result, we have now tens of thousands of gene symbols and this enormous number of abbreviations increases the probability that different genes share the same symbol created in fact from different gene names. The problem of ambiguous symbols has been raised recently [5, 6] but the issue has never been systematically studied despite its importance for developing proper publishing policies [5] and for efforts to draw meaningful conclusions from already published data [7, 8]. Therefore, we have analyzed data retrieved from the Ensembl database to identify ambiguous gene symbols in four mammalian species and tested various gene identifiers to disambiguate the gene nomenclature. We also provided current lists of ambiguous symbols and the R script to allow their extraction from the Ensembl database. Finally, the work resulted in the summary of the current state of genome annotation in analyzed species and pointed to important gaps in knowledge.

## Methods

### Summary of protein-coding genes

The lists of all protein-coding genes were downloaded from GRCh38.p14 (human genes), GRCm39 (mouse genes), mRatBN7.2 (rat genes) and Sscrofa11.1 (pig genes) datasets available in the Ensembl Genes 113 database (Ensembl BioMart genome browser www.ensembl.org/biomart/martview/ [9]). The selected filter setting was “Gene/Gene type/protein_coding” while the attributes settings (data included in the result file) was “Gene stable ID” and “Gene name” (Supplementary Fig. 1 in Supplementary file 1). The results were exported to a csv file with a default name “mart_export”. The file contains the Ensembl stable gene identifiers (Gene stable IDs) and associated gene symbols (Gene names in the Ensembl setting) or only Ensembl IDs in case of novel genes without assigned gene symbols. The file was next analyzed with an R script (R_protein-coding unique) to extract lists of unique gene symbols (exported to All_unique_protein_coding_symbols.csv file), unique gene symbols together with Ensembl stable IDs (exported to All_unique_protein_coding.csv file), gene symbols with more than one stable Ensembl ID (exported to the Ensembl_multiplied_gene_symbols.csv file) and novel genes with assigned Ensembl IDs but without a gene symbol (exported to Genes_without_gene_symbol.csv file). Additionally, the R code adds a column with gene symbols combined with the word “Gene” (for example Gene_Usp9y) to prevent an unintentional transformation of symbols by Excel. These composite identifiers can be used to check whether there are any unintentional transformation of gene symbols after uploading the data to Excel. The script used for data processing requires installation of naniar, tidyr, purrr, readr and dplyr R packages (Table 1). The list of unique gene symbols (file All_unique_protein_coding_symbols.csv) was used in the next step of the analysis aimed at identification of ambiguous gene symbols and comparison of symbols between species. The Venn diagram showing a number of overlapping gene symbols in different species was created with the script ”R_venn between species comparison” (Supplementary Fig. 1 in Supplementary file 1) employing the VennDiagram R package (Table 1). All R scripts used in this study are available in GitHub repository at https://github.com/Grzegorz-R-Juszczak/Protein-coding-gene-IDs-human-mouse-rat-pig [20] while input files for scripts are deposited at https://data.mendeley.com/datasets/454s2vw255/1 [21] in compressed folders sharing the names with the corresponding R scripts.

**Table 1:** R packages used in data analysis

*R* package	URL/Citation
tidyr	https://CRAN.R-project.org/package=tidyr [10]
dplyr	https://CRAN.R-project.org/package=dplyr [11]
purrr	https://CRAN.R-project.org/package=purrr [12]
readr	https://CRAN.R-project.org/package=readr [13]
naniar	https://CRAN.R-project.org/package=naniar [14, 15]
VennDiagram	https://CRAN.R-project.org/package=VennDiagram [16]
biomaRt	https://www.bioconductor.org/packages/release/bioc/html/biomaRt.html [17–19]

### Genomic localization of genes with multiple Ensembl IDs

The characterization of the protein-coding genes revealed striking differences between species in the number of genes possessing more than one Ensembl ID. To learn more about such genes, we retrieved their genomic localization from the Ensembl BioMart genome browser (release 113) by using the Ensembl IDs from the file Ensembl_multiplied_gene_symbols.csv generated during the previous analysis (Supplementary Figs. 1 and 2 A in Supplementary file 1). The database and datasets used for the Ensembl BioMart search were the same as in case of the summary of protein-coding genes, the filter setting was Gene/Input external references ID list/Gene stable ID(s) while the output attributes were Gene stable ID, Gene name, Chromosome/scaffold name, Gene start (bp), Gene end (bp) and Strand (Supplementary Fig. 2B in Supplementary file 1). The output file mart_export.txt was next analyzed with the R script “R_Ensembl multiplied gene symbols”. The analysis included separation of Ensembl IDs with chromosomal localization from IDs with scaffold localization (scaffolds are bioinformatic constructs constituting an intermediate step of genome assembly, https://www.ensembl.org/info/genome/genebuild/chromosomes_scaffolds_contigs.html) [22]. The list of IDs assigned to scaffolds was exported to Genes_with_scaffold_locus.csv file.

The data restricted to genes with Ensembl IDs assigned only to chromosomes were again tested for the presence of symbols with more than one Ensembl ID. The new list containing genes with more than one Ensembl ID assigned to chromosome was analyzed to identify cases when alternative Ensembl IDs were assigned to different chromosomes including at least one autosomal chromosome (file Multiplied_symbols_DifferentChromosemes_Autosomal.csv), only sex chromosomes (file Multiplied_symbols_DifferentChromosemes_Sex.csv), the same chromosome but opposite strands (file Multiplied_symbols_TheSameChromosome_Different_strand.csv), the same chromosome and start position (file Multiplied_symbols_TheSameChromosome_The_same_start.csv) and the same chromosome but different start position (file Multiplied_symbols_TheSameChromosome_Different_start.csv). Additionally, alternative Ensembl IDs assigned to a different start position at the same strand were tested for an overlap by comparing end and start positions of alternative IDs. The number of comparisons was adjusted for each species based on the maximum number of alternative Ensembl IDs assigned to a single gene symbol and the data were exported to file Multiplied_symbols_TheSameChromosome_Different_start_final.csv. The script used for data processing requires installation of dplyr, tidyr, readr, dplyr and purr R packages (Table 1).

### Summary of gene synonyms and official symbols

The list of all protein-coding genes for each species with information about gene symbols, synonyms and Ensembl IDs was downloaded from the Ensembl BioMart genome browser (release 113), using databases listed previously. The selected filter setting in the Ensembl BioMart genome browser was “Gene/Gene type/protein_coding” while the attributes settings were “Gene Synonym”, “Gene name” and “Gene stable ID” (Supplementary Fig. 3 A in Supplementary file 1). The results were exported to a csv file with a default name “mart_export” and processed with the script “R_summary gene synonyms and official symbols” to create a list of unique gene synonyms and official symbols for the Venn diagram. The first step of data processing was the creation of new columns containing gene synonyms and official gene symbols written only in lowercase letters to avoid confusion caused by a diverse usage of the uppercase and lowercase letters in gene symbols. Missing synonyms and official symbols were marked with an “NA” symbol to enable removal of such entries, and the lists of unique synonyms and official symbols were exported to Unique_gene_synonyms.csv and Unique_gene_symbols.csv files. Finally, the lists of genes were used to create the Venn diagram showing the number of gene synonyms, official symbols and the overlap between them (Supplementary Fig. 3B in Supplementary file 1) separately for each analyzed species. The script used for this analysis (R_summary gene synonyms and official symbols) requires installation of naniar, tidyr, dplyr and VennDiagram R packages (Table 1).

### Ambiguous official symbols

**Fig. 1:**
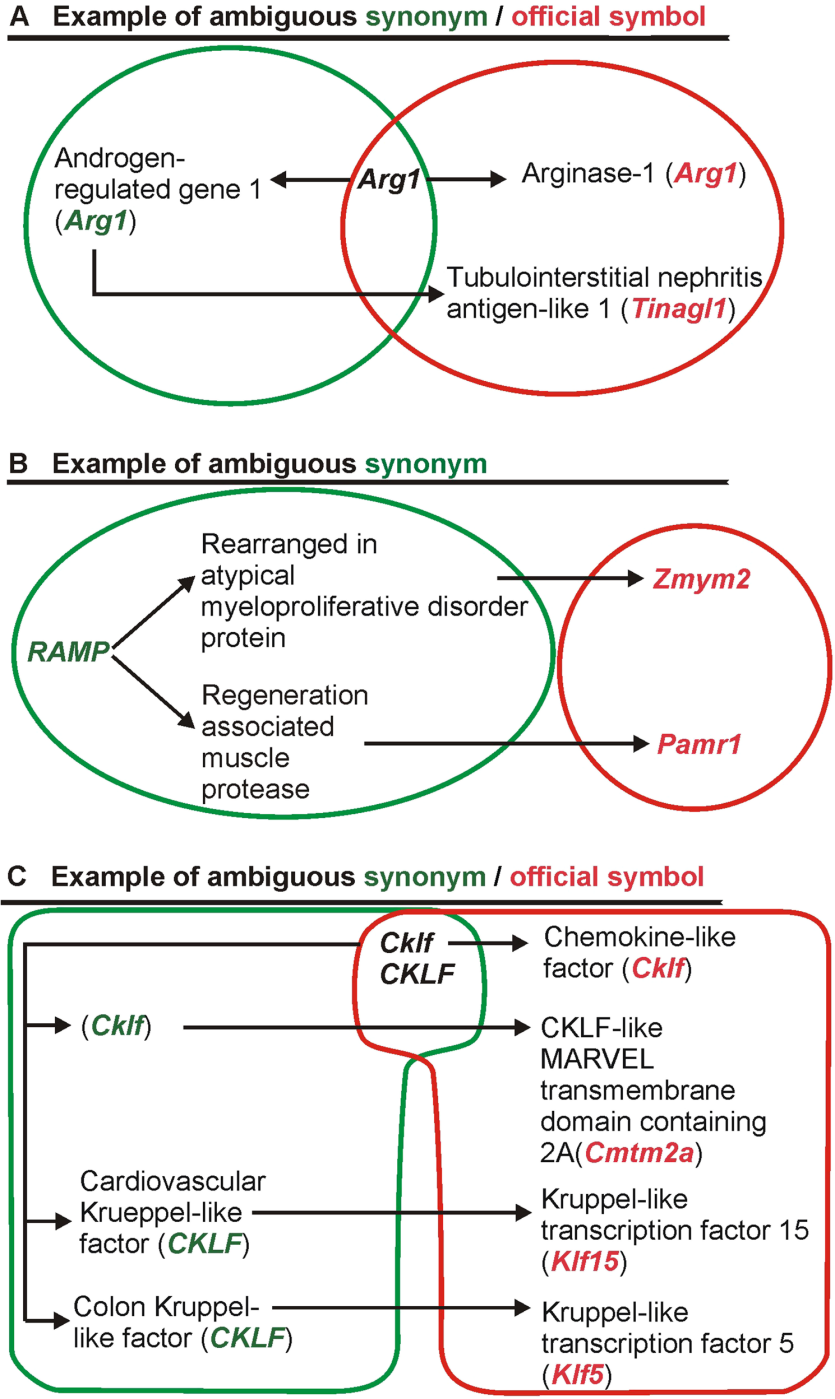
Types of ambiguous symbols with examples from the mouse genome. Green color (left side of the figure) indicates synonyms. Red color (right side of the figure) indicates official symbols. Panel **A** shows an example of ambiguous symbol *Arg1* that can mean either an official symbol derived from the name Arginase-1 or synonym derived from the name “Androgen-regulated gene 1” (current official gene symbol *Tinagl1*). Panel **B** shows an example of ambiguous synonym *RAMP* that can mean either “Rearranged in atypical myeloproliferative disorder protein” (current official symbol *Zmym2*) or “Regeneration associated muscle protease” (current official symbol *Pamr1*). Panel **C** shows an example of more complex ambiguity combining relationships between symbols presented in example **A** (ambiguous official symbol/synonym) and **B** (ambiguous synonym). *Cklf* is both an official symbol of chemokine-like factor and a synonym of three other genes with current official symbols *Cmtm2a*,* Klf5* and *Klf15*

Some symbols can be used both as a current official symbol of one gene and as a synonym for another gene (Fig. 1A). For example, *Arg1* is an official symbol of Arginase 1 and a synonym of another gene with the current official symbol *Tinagl1* (Fig. 1A). To identify such ambiguous symbols, we used the lists of current official symbols of protein coding-genes (file All_unique_protein_coding_symbols.csv) obtained in the previous analysis (Summary of protein-coding genes; Supplementary Figs. 1 and 4B in Supplementary file 1). Each genome (human, mouse, rat and pig) was searched again with the list of official symbols uploaded as synonyms in the filter panel of the Ensembl BioMart genome browser 113 (selected filter setting: Gene/Input external references ID list/Gene Synonym(s)). Selected attributes were Gene Synonym, Gene name, Gene type and Gene stable ID (Supplementary Fig. 4B in Supplementary file 1). The Gene Synonym column in the results file (mart_export.csv) contains in fact current official symbols from the previous analysis that turn out to be used also as synonyms of other genes. The gene name column contains a new list of official symbols associated with the synonyms. The remaining two columns (Gene type, Gene stable ID) contain additional information about the new official symbols from the gene name column.

The data retrieved from the Ensembl BioMart genome browser are not sufficient for identification of truly ambiguous official symbols and, therefore, require further processing that was performed with the R script “R_ambiguous official symbols” (Supplementary Fig. 4B in Supplementary file 1). In some cases there is the same symbol in the gene synonym and gene name columns suggesting that a symbol that was a synonym in the past became later an official symbol during the process of nomenclature standardization. Such cases were removed with the R script. The comparison between synonyms and official symbols was done without recognizing the uppercase and lowercase letters. This means that, for example, synonym *ADAM12* and current official symbol *Adam12* were considered as identical and were removed from the list of ambiguous symbols. Typically, the ambiguous symbols refer to different protein-coding genes but in the minority of cases one symbol can be used as an official symbol of protein-coding gene and as a synonym of another type of gene. For example, *Parp2* is the official symbol of protein coding gene with Ensembl ID ENSMUSG00000036023 and a synonym of the *Rpph1* gene on the opposite strand that generates a ribozyme (Ensembl ID: ENSMUSG00000092837). For simplicity, we focused, only on ambiguous symbols that mapped to protein-coding genes. Symbols that mapped to other gene types were removed during data processing and saved in a separate file

Ambiguous_symbols_protein_other.csv. Finally, some ambiguous official symbols/synonyms can be attributed to more than one alternative official gene symbol and, therefore, the data were grouped by gene synonym to create the final list of ambiguous gene symbols saved in the file mart_export_5.csv. The data were, next, used for the final editing of the dataset because a clearcut identification of genes labelled with ambiguous symbols requires additional information such as stable Ensembl IDs. Unfortunately, due to the restrictions imposed by the Ensembl BioMart searching tool, the retrieved data contain only IDs associated with the alternative official symbols while IDs associated with the input official symbols are missing. Therefore, the final list of ambiguous gene symbols saved in the file mart_export_5.csv was merged with the dataset saved in the All_unique_protein_coding.csv file which contains input symbols used to search the Ensembl BioMart database together with Ensembl stable IDs (Supplementary Fig. 4B in Supplementary file 1). The file All_unique_protein_coding.csv was generated during the previous analysis described in the section “Summary of protein-coding genes” while the merging of the datasets was performed with the part B of the script “R_ambiguous official symbols”. The final stage of data processing was performed with the part C of the script to edit the names of the columns and to provide additional information enabling easy identification of data type and species in the supplementary data. The part C of the script also contains the command removing unpaired input (Ambiguous_official_symbols) and output data (*tolower_Gene_Synonyms*). The lack of congruence between input and output data is a rare error caused by the occurrence of erroneous space inside of the gene symbol. We noted such a problem in case of one gene (*Igsf7 l1*, ENSRNOG00000048771) in the rat Ensembl 113 genome (corrected in Ensembl 114 release). The Ensembl BioMart genome browser (release 113) lists *Igsf7 l1* as a protein coding gene but is not recognizing it properly when the symbol is used in the filter panel, leading to a separation into two symbols (*Igsf7* and *l1*) and erroneous association with two other genes due to the existence of synonym symbol L1. Erroneous spaces, however, can also be introduced unintentionally in research data leading to misidentification of gene symbols. The edited data were saved in the Ambiguous_symbols_protein_protein_final.csv file. The output file also contains additional columns with symbols anchored to the word “Gene” (for example Gene_Ltbp2) that cannot be unintentionally altered during the data import to Excel files. The script used for this analysis requires installation of tidyr, purrr, readr and dplyr R packages (Table 1).

### Ambiguous synonyms

The analysis was performed on the data that were used previously for the summary of gene identifiers (section “Summary of gene synonyms and official symbols”). The files downloaded from the Ensembl (release 113) for each species (default mart_export.txt files) were analyzed with the R script (R_ambiguous synonyms; Supplementary Fig. 3B in Supplementary file 1) to identify ambiguous synonyms that can be assigned to at least two different official gene symbols (Fig. 1B). The first step of data processing was to remove all genes without synonym symbols. Next, the dataset was grouped by gene synonym to bring together all official symbols linked to each gene synonym. The grouping was done without recognizing the uppercase and lowercase letters in gene synonyms and resulted in the creation of a new column containing gene synonyms written only in lowercase letters. Finally, we tested whether there is more than one official symbol assigned to each synonym, and the list of ambiguous synonyms (assigned to at least two official symbols) was saved in the Ambiguous_gene_synonyms.csv file. The final stage of the data processing was performed with the part B of the script to edit the names of the columns and to provide additional information enabling easy identification of the data type and species in the supplementary files. The edited data were saved in the Ambiguous_gene_synonyms_final.csv file (Supplementary Fig. 3B in Supplementary file 1). The R script also created an additional column with gene symbols anchored to words “Gene synonym” (for example Gene_synonym_nd1) to prevent unintentional transformations of symbols by Excel. It is required to install the naniar, tidyr, purrr, readr and dplyr R packages to run the script (Table 1).

### Verification of additional gene IDs

Genes are identified not only with symbols but also with additional numeric or alphanumeric identifiers assigned by scientific organizations. Therefore, we tested different types of identifiers assigned to genes in a species-specific manner by the Ensembl, NCBI and specialized committees devoted to gene nomenclature (Table 2). The verification of IDs was composed of two steps. First, we used the results from the previous analyses to extract a complete list of all official symbols that can be affected by the symbol ambiguity. The extraction was performed with the script “R_genes linked to ambiguous symbols” separately for each species based on the previous output files Ambiguous_gene_synonyms_final.csv and Ambiguous_symbols_protein_protein_final.csv while the results were exported to the file All_genes_linked_to_ambiguous_symbols.csv (Supplementary Fig. 5 in Supplementary file 1). The list was next used to extract associated IDs from the Ensembl 113 genome datasets described in the section “Summary of protein-coding genes”. The selected filter setting was “Gene/Input external references ID list/Gene Name(s)” while the attributes in Gene/Ensembl and External/External references directories were “Gene name”, “Gene stable ID”, “NCBI gene (formerly Entrezgene) ID” and one of the IDs provided by the nomenclature committee that is mouse “MGI ID”, rat “RGD ID”, human “HGNC ID” and pig “VGNC ID”. The results were exported to the text file mart_export and processed with scripts adjusted for individual species (R_test of additional IDs in Ensembl Mouse/Human/Rat/Pig). The scripts identified missing IDs and IDs assigned to more than one official gene symbol. The results were exported to the files “Missing_IDs.csv”, “Multiplied_Ensembl_IDs_final.csv”, “Multiplied_NCBI_IDs_final.csv” and “Multiplied_Committee_IDs_final.csv”. It is required to install the naniar, tidyr, dplyr and readr R packages to run the script (Table 1).

**Table 2:** Reference databases used to retrieve gene symbols and stable gene IDs supporting gene identification. Ensembl – a sequence-centered bioinformatic project of the European Molecular Biology Laboratory [23, 24]; NCBI – the National Center for Biotechnology Information [25]; MGI - Mouse Genome Informatics [26]; RGD - Rat Genome Database [27]; HGNC - HUGO Gene Nomenclature Committee [28]; VGNC - Vertebrate Gene Nomenclature Committee [29]

Database	URL/file name if multiple files are available/citation	Species	Transthyretin gene ID
Ensembl	www.ensembl.org/biomart/martview/ [9]	Multiple	ENSMUSG00000061808 (mouse ID)
NCBI	www.ncbi.nlm.nih.gov/datasets/genome/ [30] (data for specific genome assemblies) https://www.ncbi.nlm.nih.gov/datasets/gene/ [31] (data only for species)	Multiple	22139 (mouse ID)
MGI	https://www.informatics.jax.org/downloads/reports/index.html [32]/MRK_List1.rpt, MRK_List2.rpt and MRK_ENSEMBL.rpt files	Mouse	MGI:98865
RGD	https://download.rgd.mcw.edu/data_release/RAT/ /GENES_RAT.txt file/[27, 33]	Rat	3916
HGNC	https://www.genenames.org/ [34] HGNC gene symbol reports, HGNC BioMart tool	Human	HGNC:12405
VGNC	https://vertebrate.genenames.org/download/statistics-and-files/ [35]/pig_vgnc_gene_set_All.txt file	Multiple	VGNC:94573 (Pig ID)

Missing IDs and IDs assigned to more than one official gene symbol in the Ensembl genome browser were finally checked in the genome data retrieved from the proprietary databases listed in Table 2. Data verification and final editing were performed with the scripts “R_NCBI test”, “R_RGD test”, “R_HUGO test” and “R_vgnac test” following the procedures used in the previous sections. The results exported to csv files (Verification_missing_NCBI_IDs.csv, Summary_NCBI_IDs_ambiguous_in_Ensembl.csv, Summary_RGD_IDs_ambiguous_in_Ensembl.csv, Verification_missing_HUGO_IDs.csv and.

Verification_missing_VGNC_IDs.csv) were used for the preparation of supplementary data. The complete workflow is shown by the example of verification of mouse NCBI IDs (Supplementary Fig. 5 in Supplementary file 1).

### Ensembl genes without a symbol

Differences between genomic databases prompted us to verify genes without an assigned symbol in the Ensembl database. During the first step, we used a list of Ensembl IDs from the file Genes_without_gene_symbol.csv (obtained during the initial characterization of the protein-coding genes; Supplementary Figs. 1 and 6 A in Supplementary file 1) to retrieve additional IDs (Table 2) from the Ensembl genome datasets (release 113) described in the section “Summary of protein-coding genes”. The selected filter setting (Supplementary Fig. 6B in Supplementary file 1) was “Gene/Input external references ID list/Gene stable ID(s)” while the attributes were “Gene name”, “Gene stable ID”, “NCBI gene (formerly Entrezgene) ID” and one of the IDs provided by the nomenclature committee that is mouse “MGI ID”, rat “RGD ID”, human “HGNC ID” and pig “VGNC ID” (selected in external references). The results were exported to the text file as described previously. Next, we retrieved genomic data for all genes available in alternative databases (Table 2) separately for each species. The need for inclusion of Ensembl IDs forced us to modify the data collection compared with the previous section with the exception of rat RGD and pig VGNC data. In case of NCBI data (Table 2), we used the Gene database (instead of Genome database) because it allows the inclusion of Ensembl IDs for each analyzed species but without a possibility to download data for specific genome assemblies that are provided in the Ensembl. In case of mouse MGI data, we downloaded the MRK_List2 and MRK_ENSEMBL files (Table 2) that were merged together. Finally, in case of HGNC data (Table 2), we used the HGNC BioMart tool instead of HGNC gene symbol reports (available in gene data) to satisfy the requirements of this analysis. The data from alternative resources were additionally screened for Ensembl stable IDs used as gene symbols and such entries were removed (rat RGI data). Finally, the data retrieved from the Ensembl were merged with the data collected from the other sites according to external IDs available in the Ensembl and Ensembl IDs provided in alternative databases (Supplementary Fig. 6B in Supplementary file 1). This work was performed with the script “R_Ensembl novel genes” (adjusted for each species) following the procedures used in the previous sections. Integrated data were exported to Ensembl_MGI_NCBI_grouped.csv, Ensembl_RGD_NCBI_grouped.csv, Ensembl_HGNC_NCBI_grouped.csv and Ensembl_VGNC_NCBI_grouped.csv files respectively for mouse, rat, human and pig data.

### Updating gene symbols in Ensembl

**Fig. 2:**
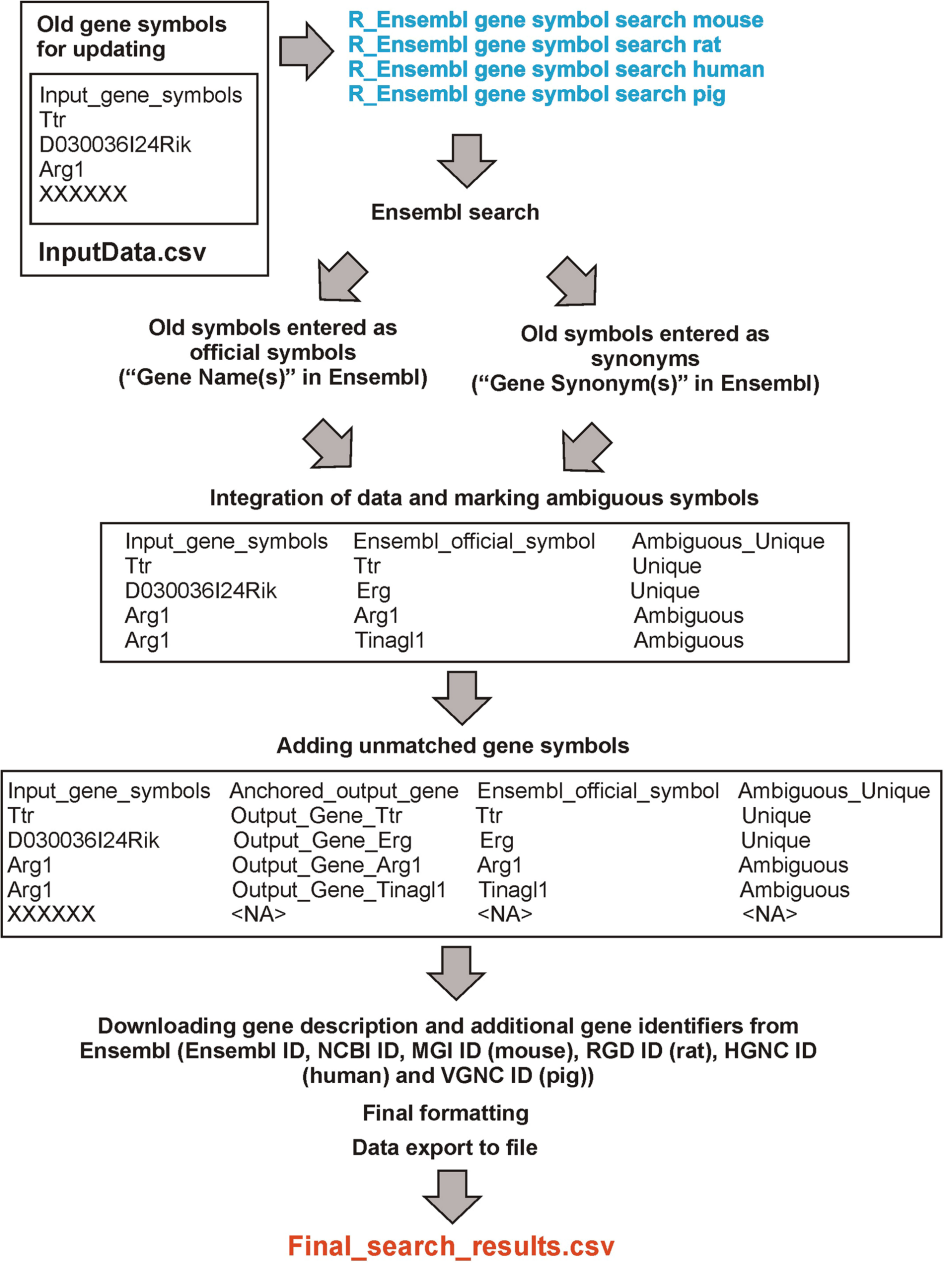
Summary of procedure used for gene symbol update performed with scripts ”R_Ensembl gene symbol search” (REgeness) adjusted for mouse, rat, human and pig data. The scripts are available at https://github.com/Grzegorz-R-Juszczak/Protein-coding-gene-IDs-human-mouse-rat-pig [20]

Each gene has a different history of scientific investigation and associated assignment of gradually refined names. Research literature contains a mixture of official gene symbols and obsolete symbols (synonyms). Not only that, but changes in gene nomenclature over time can produce new official gene symbols and turn the old official gene symbols into synonyms. Therefore, genes retrieved from the literature should be updated to reflect current official symbols to allow the reliable interpretation of data. However, updating gene symbols without the help provided by additional IDs listed in Table 2 is a complex task because some symbols yield results only when they are entered into Ensembl BioMart as official symbols, and some symbols yield results only when they are entered as synonyms while others give different results depending on the search approach. To facilitate the updating of gene symbols retrieved from literature without stable IDs, we prepared a script “R_Ensembl gene symbol search” (REgeness) that imports list of gene symbols and performs a double Ensembl search for current official symbols followed by data integration, identification of ambiguous symbols and downloading additional information and IDs in a species-specific manner (Fig. 2). The scripts combine the R code used in the previous sections with the biomaRt package enabling programmatic access to Ensembl BioMart [17, 36]. The data requirement is a list of gene symbols in the csv file named InputData with one column named Input_gene_symbols (Fig. 2). The list of current official symbols together with information about their ambiguity, gene description and additional IDs (Table 2) is exported to the Final_search_results.csv file. Additionally, the script imports and saves biomaRt codes for selecting Ensembl databases (file EnsemblDatabases.csv), datasets (EnsemblDatasets.csv), filters (file EnsemblFilters.csv) and output data called attributes in Ensembl (EnsemblAttributes.csv). These files contain information about version of Ensembl used for data downloading (file EnsemblDatabases.csv) and enable code modifications including species (EnsemblDatasets.csv) and output data associated with updated genes (file EnsemblAttributes.csv). Replacing the code for pig genome (sscrofa_gene_ensembl) with a code for other genomes listed in the EnsemblDatasets.csv file will enable gene symbol standardization for other species together with downloading associated IDs including VGNC ID assigned to 32 vertebrate species. Running the script requires installation of the biomaRt, tidyr, dplyr, purrr and readr R packages (Table 1). Problems with running the biomaRt package may result either from obsolete software or from malfunctioning of the Ensembl servers. More information about biomaRt troubleshooting is described in Supplementary file 2.

## Results

### Summary of protein-coding genes

**Fig. 3:**
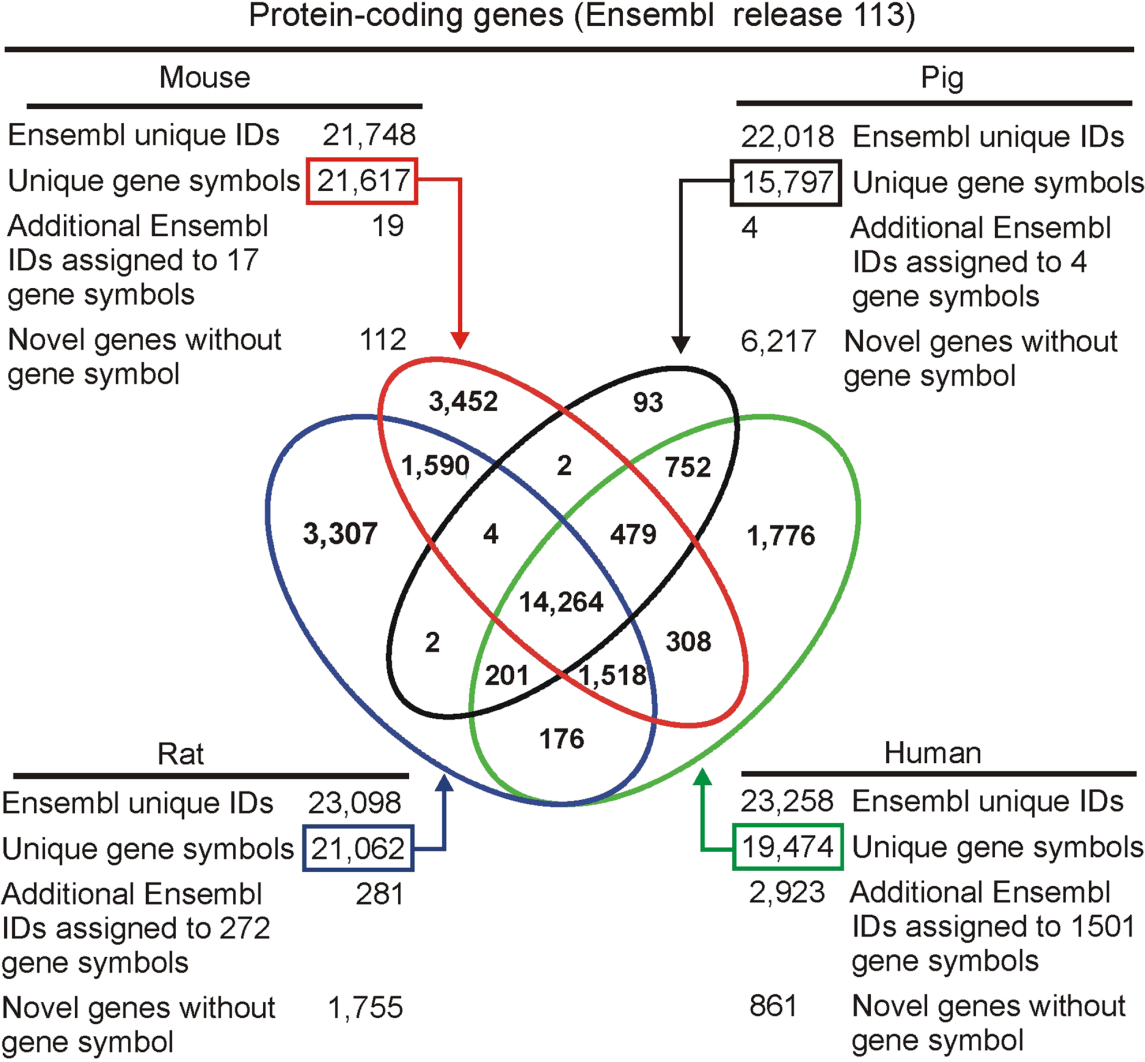
Summary of protein-coding genes in Ensembl. The Venn diagram shows the number of unique official gene symbols that are either species-specific or are common for analyzed species. Ensembl IDs – stable gene identifiers that begin “ENSG” such as the *Ttr* example in Table 2

The data retrieved from the Ensembl BioMart genome browser showed that the number of protein-coding genes with distinct Ensembl IDs ranged from 21,748 in mice to 23,258 in humans (Fig. 3). The analysis of the data revealed, however, several differences between species. First, the species differ in the number of genes with distinct symbols that is highest in mice (21,617) and rats (21,062) and lowest in pigs (15,797). Second, the genomes differ in the number of genes with an assigned unique Ensembl ID without any symbol that constitute only 0.5% of all genes in mice, 3.7% in humans, 7.6% in rats and 28.2% of genes in pigs (Fig. 3). Finally, the Ensembl gene sets differ in the number of gene symbols that can be assigned more than one stable Ensembl ID. Typically, one gene symbol is associated with only one stable Ensembl ID but in some cases it is possible to map two or, less frequently, three stable IDs to a single gene symbol. In humans, there are 1,501 such gene symbols with multiple stable IDs compared with 17 in mice and 4 in pigs (Fig. 3). These striking differences inspired the retrieval of information about genomic localization of genes with multiple Ensembl IDs. These data (Table 3) revealed that the large number of such genes in humans results from the assignment of Ensembl IDs to scaffolds that are bioinformatic constructs constituting an intermediate step in genome assembly. Therefore, the number of symbols with multiple Ensembl IDs in humans decreased to only 29 after restricting the dataset to IDs with chromosomal localization (Table 3). The data also showed that alternative Ensembl IDs assigned to the same gene display a large variability in localization that ranges from partly overlapping positions on the same chromosome to localization assigned to different strands or even different chromosomes (Table 3, examples in Table 4).

**Table 3:** Summary of gene symbols with assigned more than one Ensembl ID (Ensembl release 113)

		Gene symbols with more than one Ensembl ID			
		Mouse	Rat	Human	Pig
Ensembl IDs	assigned both to chromosomes and scaffolds	17	272	1,501	4
	assigned only to chromosomes	17	269	27	2
	assigned to sex chromosomes X and Y	0	0	18	1
	assigned to different chromosomes including at least one autosomal chromosome	0	68	0	0
	assigned to the same chromosome but different strand	0	56	0	0
	with non-overlapping localizations on the same chromosome and strand	2	95	0	1
	with overlapping localizations	15	37	9	0
	with the same localization	0	13	0	0

**Table 4:** Examples of genes with multiplied Ensembl IDs (A-C) and with Ensembl ID without assigned gene symbol (D) in Ensembl Release 113. One of the two Gcat copies with overlapping coordinates (A) is classified as an overlapping readthrough gene, according to the information provided in the Ensembl annotation attributes. The Pakap gene (B) comprises two non-overlapping sequences, ENSMUSG00000090053 and ENSMUSG00000038729, which are connected by an overlapping readthrough gene, ENSMUSG00000089945, according to the Ensembl annotation attributes. The double copy of the human TUBB gene (C) is an example of a gene that it found both on a standard chromosome and a scaffold. The unnamed mouse gene ENSMUSG00000121905 (D) was identified in Ensembl Release 111 (2024) as a member of the S100 calcium binding protein family and has remained listed as a novel protein without an assigned gene symbol in subsequent database releases, including Release 113 (the history of the gene is available at https://www.ensembl.org/info/website/archives/index.html [37])

Gene examples		Official gene symbol	Ensembl ID	Chromosome/scaffold name	Gene start (bp)	Gene end (bp)
A	Multiplied IDs with overlapping genomic position	Gcat	ENSMUSG00000006378	15	78,915,074	78,922,553
		Gcat	ENSMUSG00000116378		78,915,101	78,926,731
B	Multiplied IDs with non-overlapping genomic position	Pakap	ENSMUSG00000090053	4	57,434,247	57,712,016
		Pakap	ENSMUSG00000038729		57,717,657	57,896,984
C	Multiplied IDs with genomic position assigned to chromosome and scaffold	TUBB	ENSG00000196230	6	30,717,435	30,725,538
		TUBB	ENSG00000224156	HSCHR6_MHC_APD_CTG1	2,049,704	2,054,929
D	Gene without symbol	**-----**	ENSMUSG00000121905	3	90,822,180	90,824,241

### Gene symbols

**Fig. 4:**
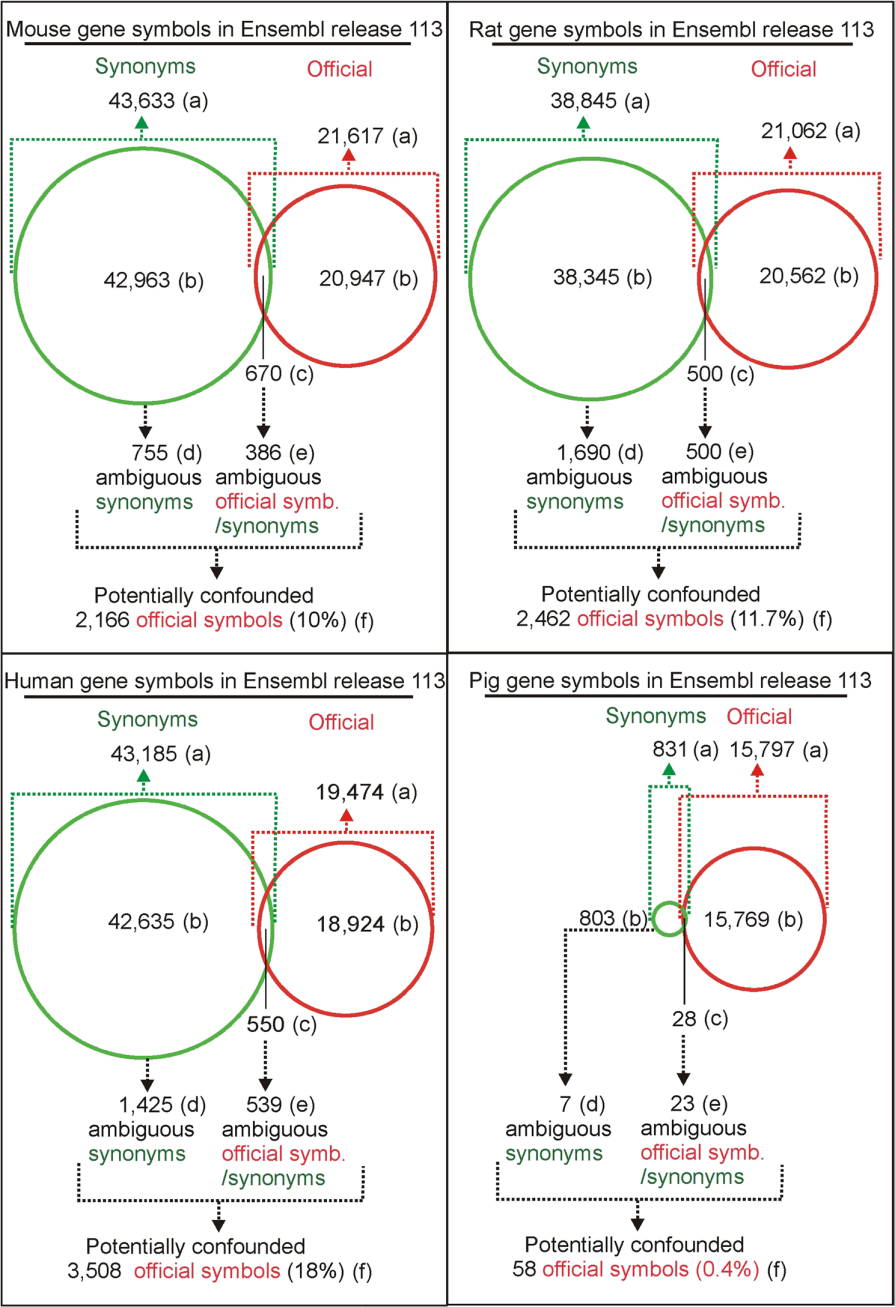
Summary of gene symbols used to identify protein-coding genes in Ensembl. **a** the total numbers of synonyms and official symbols. **b** the number of symbols unique to either the group of synonyms or the group of official symbols. **c** total number of symbols that are both synonyms and official symbols, including cases when symbols are mapped to the same gene (for example synonym *ADAM12* and current official symbol *Adam12*) or to different genes (examples in Fig. 1AC). **d** number of symbols unique to the group of synonyms that are mapped to different genes (example in Fig. 1B). **e** number of symbols that are both official symbols and synonyms mapped to different genes (examples in Fig. 1AC). **f** the total number of genes that can be misidentified due to symbol ambiguity. The number was derived from the data generated by the script “R_genes linked to ambiguous symbols”, which integrates the sets of ambiguous official symbols/synonyms and ambiguous synonyms to produce a combined list of all official symbols from both datasets

The total number of official symbols and synonyms ranged from 16,600 in pig genome to 64,580 in the mouse gene set (Fig. 4). In mouse, rat and human genomes there are approximately twice as many synonyms as official symbols in contrast to pig genome containing only a small number of synonyms (Fig.4 ). Importantly, some of the synonyms are ambiguous because they can be attributed to more than one official symbol as shown in Fig. 1B. Such ambiguous synonyms were found in all analyzed genomes but their contribution to the total number of genes varies greatly between species. The analysis found the largest number of synonyms that mapped to more than one gene in rat (1,690) while the lowest number was found in the pig gene set (7) corresponding with a very small number of synonyms in this species (Fig. 4). There are also some ambiguous symbols that are both synonyms and official symbols (Figs. 1A and 4). The number of official symbols that are at the same time synonyms in other genes ranges from 539 in the human gene set to only 23 for pig (Fig. 4). The list of all ambiguous symbols in each species is provided in Supplementary file 3.

However, assessing the impact of these symbols on gene identification is not straightforward. On the one hand, the impact of ambiguous symbols can be greater than their number because, in principle, each maps to multiple genes. The examples in Fig. 1 show that the three symbols (*Arg1*, *Ramp*, and *Cklf*) can be linked to eight genes, indicated by the red gene symbols on the right side of the figure. On the other hand, the total number of potentially misidentified genes may be lower than the number of ambiguous symbols, because multiple synonyms can be mapped to a smaller set of common genes. For example, four ambiguous rat synonyms, *c-H-ras*, *H-Ras-1*, *HRAS1*, and *p21ras*, are mapped to the same set of two genes, *Hras* and *Nras*. Therefore, the total number of genes that can be misidentified due to symbol ambiguity was calculated based on the results generated by the script “R_genes linked to ambiguous symbols”, which integrates the sets of ambiguous official symbols/synonyms (examples in Fig. 1A–C) and ambiguous synonyms (example in Fig. 1B) to produce a combined list of all official symbols from both datasets. Integration of the datasets revealed that the number of genes potentially misidentified due to symbol ambiguity (values marked with (f) in Fig. 4) is greater than the sum of ambiguous symbols (marked with (d) in Fig. 4) and ambiguous official symbols/synonyms (marked with (e) in Fig. 4) in all analyzed species. The integrated datasets showed that approximately 10% of protein-coding genes in laboratory rodents and 18% of human genes (Fig. 4) have synonyms that can be mapped to more than one gene (example in Fig. 1B) or have official symbols that are also used as synonyms mapped to other genes (examples in Fig. 1A–C).

### Disambiguation of symbols with additional gene IDs

Genes can be identified not only with symbols but also with stable IDs assigned by Ensembl, NCBI and specialized committees (Table 2). Therefore, we used the Ensembl BioMart genome browser to check the availability of these IDs and their utility for disambiguation of gene symbols. The IDs were retrieved for each analyzed species using the lists of all official symbols that can be confused due to gene symbol ambiguity. As expected, all problematic gene symbols identified previously in the Ensembl database have unique Ensembl IDs allowing for their disambiguation. Furthermore, the data showed that most of these genes also have other identifiers available in the Ensembl database, with some exceptions. For example, Ensembl did not provide NCBI IDs for nine mouse genes, 359 rat genes, and one human gene (Supplementary file 4). The verification of gene symbols directly in the NCBI genome database (human GRCh38.p14, mouse GRCm39, and rat mRatBN7.2 genomes; www.ncbi.nlm.nih.gov/datasets/genome/ [30]) showed that the majority of these genes are also included in the NCBI genomes and have assigned NCBI IDs (Supplementary file 4). In these cases, the absence of NCBI IDs in Ensembl resulted from gaps in information exchange between the databases. The remaining Ensembl gene symbols lacking NCBI IDs were not found in the NCBI database (Supplementary file 4), indicating differences between major databases in genome annotation. A small number of genes without additional IDs in Ensembl was also found in case of identifiers assigned by specialized committees HGNC (2 genes) and VGNC (20 genes). Most of these cases resulted from the lack of corresponding gene symbols in the proprietary databases (Supplementary File 4), again indicating differences in genome annotation.

Finally, we have checked whether the IDs assigned by Ensembl, NCBI and specialized committees (Table 2) allow for disambiguation of official symbols that can be confused due to their association with ambiguous synonyms. The identification of missing IDs and IDs assigned to more than one official gene symbol in the Ensembl database was performed using the script “R_test of additional IDs in Ensembl”. Consistent with expectations, the data retrieved from the Ensembl database showed an unequivocal association between all gene symbols and Ensembl IDs for all tested species and also unequivocal association between majority of gene symbols and mouse MGI IDs, human HGNC IDs, pig VGNC IDs and pig NCBI IDs. The exceptions applied to 1 mouse NCBI ID, 42 human NCBI IDs, 53 rat NCBI IDs and 645 rat RGD IDs that were assigned to more than one gene symbol in the Ensembl database (Supplementary data 4). For example, Ensembl BioMart assigned the same NCBI ID 100040048 to two different mouse genes, Ccl27a and Ccl27b (Supplementary data 4). However, the data retrieved directly from the proprietary NCBI and RGD databases did not confirm these ambiguous associations found in Ensembl. For example, the NCBI assigns the aforementioned ID 100040048 only to one mouse gene, Ccl27b (Supplementary data 4).

### Ensembl genes without symbols

The analysis of protein-coding genes revealed large differences in the number of Ensembl novel genes with assigned Ensembl stable IDs but without gene symbols, (Fig. 3) pointing to important gaps in understanding some genes. However, differences between genomic databases prompted us to check the status of these genes in other major databases using NCBI, MGI (mice), RGD (rat), HGNC (human) and VGNC (pig) IDs available in Ensembl and Ensembl IDs available in other databases. The comparison between databases revealed that some gene symbols missing in Ensembl are available in other databases. The inspection of the data revealed, however, that some identifiers included in the gene symbol columns are in fact NCBI “LOC” gene identifiers composed of prefix LOC that is followed by NCBI numeric gene ID. Such identifiers are temporary assigned to genes lacking a true symbol and indicate lack of established gene nomenclature (https://www.ncbi.nlm.nih.gov/books/NBK3840/#genefaq.Nomenclature [38]). Therefore, genes assigned only to “LOC” identifiers were not counted as having a gene symbol. The final data are presented in Table 5. Although subtraction of genes with symbols available in alternative databases (NCBI, MGI, RGD, HGNC and VGNC) resulted in the lower number of Ensembl genes without gene symbols, the corrected data still indicated large differences between species in genome annotation (Table 5).

**Table 5:** Number of novel protein-coding genes without gene symbols in the Ensembl database (release 113) and novel Ensembl genes with assigned gene symbols in other databases. Numbers in parentheses indicate Ensembl novel genes with more than one gene symbol assigned in an alternative database

Species	Number of genes						
	Ensembl	NCBI	MGI	RGD	HGNC	VGNC	Corrected Ensembl
Mouse	112	12 (2)	0	-	-	-	100
Rat	1,755	2 (1)	-	1	-	-	1,752
Human	861	57 (2)	-	-	3	-	801
Pig	6,217	1,038 (9)	-	-	-	591	5,082

### Updating gene symbols in Ensembl

The R scripts R_Ensembl gene symbol search (REgensess) were prepared in variants adjusted for mice, rats, humans and pigs. The scripts were tested on trial sets of gene symbols containing both unique and ambiguous official symbols and synonyms selected from the data obtained in the previous sections. Moreover, we added a nonsense symbol XXXXXX to control the proper processing of unmatched data. The rat dataset also contained the Igsf7 l1 symbol with an erroneous space present in Ensembl release 113 (corrected in subsequent release 114). The lists of trial symbols together with additional descriptions are provided in Supplementary file 5 while the output files Final_search_results.csv from symbol update for individual species are provided in Supplementary files 6–9. The script provided results that were expected based on an online Ensembl BioMart search with the exception of the single rat gene with erroneous space that is recognized during the online search as two symbols leading to the assignment of ambiguous matches. This problem, however, was not encountered when the data were downloaded with biomaRt package implemented in the REgensess script that provides exactly the same gene in output data (input symbol *Igsf7 l1*, output symbol *Igsf7 l1*).

Additionally, we compared our script with two online search tools that enable the updating of symbols. The online search was performed on 14 and 15 January 2025 with mouse trial genes (Supplementary file 5) and results are provided in Table 6. The bioDBnet [39] and GeneToList [6] tools generated partially overlapping result. Close inspection of the bioDBnet tool revealed that it finds matches that are not in the Ensembl database but are recorded by NCBI (*Dtl* as an ambiguous match for input symbol *Ramp).* Furthermore, bioDBnet is case sensitive, retrieving different results depending on the usage of uppercase and lowercase letters. As a result, search for *Cklf* and *CKLF* yields different results (either *Cklf* and *Cmtm2a* o*r Klf15* and *Klf5)* while our script is case insensitive and lists all four symbols irrespective of the usage of lowercase and uppercase letters. The case sensitivity also means that any changes in symbol lettering that are different from official guidelines block the recognition of symbols by the bioDBnet tool. For example, bioDBnet is not providing any results for mouse symbols *ttr* (official *Ttr*) and *D030036I24rik (D030036I24Rik)* while such symbols are recognized during the Ensembl search. Another set of differences were found in case of the GeneToList online tool (Table 6). First, it is ignoring alternative matches when one perfect match is available as indicated by the *Cklf* and *Arg1* symbols. Second, it suggests genes with an exact synonym match (genes *Zmym2*, *Pamr1*, *Dtl* from common synonym *Ramp*) and genes with a partial synonym overlap (shown in bold) such as *Serp1* (synonym ***Ramp****4*), *Bin1* (synonym ***RAMP****−2*), *Tram1* (synonym *T****RAMP***) and *Tnfrsf25* (synonym *T****RAMP***). The users are also asked to select one match from the list of suggested matches to create the final list of updated symbols.

**Table 6:** Comparison between tools allowing updating gene symbols. REgeness – R_Ensembl gene symbol search script developed in this study. bioDBnet – https://biodbnet-abcc.ncifcrf.gov/db/db2db.php [39, 40]. GeneToList – https://www.genetolist.com/ [6, 41]

Input trial symbols	Updated gene symbol			Verification in Ensembl	Verification in NCBI
	REgeness	bioDBnet	GeneToList		
*Ttr*	*Ttr*	*Ttr*	*Ttr*	Positive	Positive
*Gcat*	*Gcat*	*Gcat*	*Gcat*	Positive	Positive
*D030036I24Rik*	*Erg*	*Erg*	*Erg*	Positive	Positive
*Adam12*	*Adam12*	*Adam12*	*Adam12*	Positive	Positive
*Arg1*	*Arg1*	*Arg1*	*Arg1*	Positive	Positive
	*Tinagl1*	*Tinagl1*	NA	Positive	Positive
*Ramp*	*Zmym2*	NA*	*Zmym2*	Positive	Positive
	*Pamr1*	*Pamr1*	*Pamr1*	Positive	Positive
	NA	*Dtl*	*Dtl*	Negative	Positive
	NA	NA	Serp1**	Negative	Negative
	NA	NA	Bin1**	Negative	Negative
	NA	NA	Tram1**	Negative	Negative
	NA	NA	Tnfrsf25**	Negative	Negative
*Cklf*	*Cklf*	*Cklf*	*Cklf*	Positive	Positive
	*Klf5*	NA*	NA	Positive	Positive
	*Klf15*	NA*	NA	Positive	Positive
	*Cmtm2a*	*Cmtm2a*	NA	Positive	Positive

*NA* Not available in search results, NA* Not available in search results because the tool is case sensitive which means that for example symbol Cklf and CKLF give different results

**Genes listed as suggested matches with partly overlapping synonym symbols indicated with bold letters in online service

## Discussion

The study provides a detailed overview of Ensembl protein-coding genes and explains relationships between various gene identifiers assigned by Ensembl, NCBI and specialized committees such as MGI, RGD, HGNC and VGNC (Table 2). The most important finding is that a large number of gene symbols in the most frequently studied species, laboratory rodents and humans, can map to more than one gene, either via the official gene symbol or via a synonym (Fig. 4). Symbols that map to more than one gene may lead to confusion in 10% of rat and mouse genes and 18% of human genes, as indicated by the estimate based on the protein-coding genes in the Ensembl database (Fig. 4).

Our results also indicated that the impact of the symbol ambiguity is even larger after inclusion of other types of genes. Therefore, it constitutes much more severe problem than previously reported transformations of some gene symbols caused by Excel [1–4]. The misidentifications caused by symbol ambiguity are most likely in case of literature data retrieved from older studies using past versions of gene nomenclature with a large number of obsolete symbols. A simple solution for this problem is to use stable gene IDs (Table 2) for unequivocal identification of genes. Gene symbols derived from abbreviated full gene names are convenient because they convey functional meaning and can be easily memorized while the stable IDs enable disambiguation of gene nomenclature. Therefore, reporting both gene symbols and stable IDs is a best solution combining advantages of different naming systems.

It is important, however, to retrieve genomic information associated with gene identifiers directly from proprietary databases hosted by organizations responsible for the assignment of these IDs. Such proprietary databases contain the most accurate data that are not affected by a delayed exchange of information between databases due to different time schedules of data updates. Furthermore, genomic databases are not fully compatible due to differences in genome annotation caused by the usage of different raw sequence data, different methodology and unusual complexity of some loci that cannot be easily fitted in the canonical view of a gene [36]. The existence of such discrepancies is another reason for using proprietary databases for each type of gene identifiers (Table 2).

Although identifiers listed in Table 2 should be used as a first choice for updating genomic data retrieved from literature, there are many instances when only gene symbols are available. Such data contain both symbols that are still considered as official and obsolete symbols that are now classified as gene synonyms (aliases). Such literature datasets containing only gene symbols are difficult to update and are prone to errors. Therefore, we developed the R script REgeness that imports the list of gene symbols and performs Ensembl search for current official symbols followed by data integration, identification of ambiguous symbols and downloading additional information and IDs in a species-specific manner (Fig. 2). The comparison with other available tools (Table 6) shows that even such discrete changes as differences in the usage of uppercase and lowercase letters in gene symbols may affect results of gene updating leading to different results or preventing recognition of searched symbols. The usage of uppercase and lowercase letters in symbols is regulated by specialized nomenclature committees and varies between species and named molecules with the distinction between genes and proteins (https://www.genenames.org/about/guidelines/ [42], https://www.informatics.jax.org/mgihome/nomen/gene.shtml [43]). Nomenclature policies also changed over time and include exceptions from general rules. As a result, the format of gene symbols can be easily confused by authors or altered by software used for data processing, editing and visualization while the correction of uppercase and lowercase letters in larger datasets is prone to errors due to various exceptions. Therefore, our script REgeness developed for gene name standardization (Fig. 2; Table 6) is letter case insensitive similarly to the Ensembl BioMart genome browser but in contrast to the bioDBnet [39] online tool (Table 6). The script also applies a consistent approach for indicating ambiguous symbols in contrast to the GeneToList online tool [6] that skips alternative symbols when a perfect match between an old symbol and a current official symbol is available (Table 6). Finally, the other advantages of the R script, as compared with online tools, are transparency of the procedure that can be traced in the publicly available R code, replicability of data processing, and flexibility because the script can be modified including the selection of various data available in the Ensembl BioMart. Therefore, the R script constitutes a valuable tool for updating gene symbols.

The second most interesting finding is a large disparity between pigs and other analyzed species in the number of gene symbols and novel protein-coding genes without assigned symbols. Ensembl provides only 15,797 pig gene symbols compared with 21,617 mouse gene symbols, and 6,217 novel genes in pig genome compared with only 112 mouse genes without assigned gene symbols (Fig. 3). These numbers can be corrected by comparison with other databases but even the revised data show large differences between species (Table 5). This indicates a large gap in understanding protein-coding genes in pigs that are less frequently studied than humans, rats and mice but have anyway an important economic [44, 45] and medical impact [46, 47]. Surprisingly, it is even difficult to compare the total number of protein-coding genes between species because of the differences in genome annotation, uncertain status of novel genes without assigned gene symbols and not straightforward relationship between gene symbols and Ensembl stable IDs that can lead to overestimation of the number of genes (Fig. 3; Table 3). What is certain, however, there is much more work to be done to understand pig genome and to make reliable comparisons between species.

Data available in genomic databases will undergo gradual changes due to scientific progress, continuing standardization efforts and exchange of information between databases. Therefore, we provided R scripts enabling extraction of specific information also from updated databases. It should be noted, however, that various aspects presented in this paper are expected to change in a different way and at different paces. For example, the number of ambiguous gene symbols that are grounded in literature data will not decrease and therefore, will pose a continuing challenge for integration of genomic data derived from different studies. On the other hand, the number of new genes lacking gene symbols will diminish due to scientific progress. The search of the newest version of the Ensembl database shows that 85 pig genes lacking symbol in the Ensembl release 113 have assigned gene symbol in Ensembl release 114 while none such change occurred in case of mouse genes. This indicates the rate of changes ranging from 0% (mouse) to 1.4% (pig) of novel genes between consecutive versions of the Ensembl database. Therefore, the rate of changes is relatively slow but detectable. Similarly, some errors that constitute an inevitable part of each scientific process are corrected in updated versions of genomic databases. For example, the rat gene symbol *Igsf7 l1 (*ENSRNOG00000048771*)* with erroneous space found in Ensembl release 113 is corrected in subsequent release 114 and appears with a different Ensembl ID (ENSRNOG00000046216) assigned previously to gene *Igsf7* that is now withdrawn. This case also illustrates challenges associated with annotation of some genes. Importantly, changes between current and past releases of Ensembl can be traced in archival data deposited at https://www.ensembl.org/info/website/archives/index.html [37] to support the proper interpretation of older data. It should be noted, however, that reliable updates are possible only in case when research data contains stable gene IDs (Table 2) that should accompany gene symbols. Therefore, the work performed by Ensembl and other organizations (NCBI, MGI, RGD and VGNC) that analyze genomes, assign gene IDs and provide large-scale datasets is crucial for life sciences which rely heavily on genomic data.

## Conclusions

In the mouse, rat and human genomes, there are approximately twice as many synonyms (aliases) as official symbols. This large number of obsolete gene symbols leads to the problem with unequivocal identification of genes in the literature data because some synonyms can be attributed to more than one current official symbol of protein-coding genes. The ambiguity of symbols may lead to misidentification of 10% of rodent genes and even 18% of human protein-coding genes. Such misidentifications are most likely in case of literature data retrieved from older studies using past versions of gene nomenclature with a large number of obsolete symbols. A simple solution for this problem is usage of stable gene IDs (Table 2) for the unequivocal identification of genes, provided that the genomic information associated with these IDs is retrieved directly from proprietary databases containing the most accurate data. The usage of stable identifiers is important not only for accurate interpretation of literature data but also for currently published datasets because neither the annotation of genomes nor the understanding of gene function are complete.

This incompleteness means that official gene symbols will change over time. Data based only on gene symbols should be used cautiously to avoid misidentification of genes. A solution for this problem is our R script that updates gene symbols and provides annotation about their unique or ambiguous character.

## Supplementary Information

Supplementary Material 1.

Supplementary Material 2.

Supplementary Material 3.

Supplementary Material 4.

Supplementary Material 5.

Supplementary Material 6.

Supplementary Material 7.

Supplementary Material 8.

Supplementary Material 9.

## Data Availability

All raw data used in this study are available in public databases indicated in the manuscript (Ensembl, NCBI Genome, NCBI Gene, MGI, RGD, HGNC and VGNC). Processed data are provided in supplementary files. R code used to analyze the data is available at [https://github.com/Grzegorz-R-Juszczak/Protein-coding-gene-IDs-human-mouse-rat-pig](https://github.com/Grzegorz-R-Juszczak/Protein-coding-gene-IDs-human-mouse-rat-pig).The input data for the R scripts are available at [https://data.mendeley.com/datasets/454s2vw255/1](https://data.mendeley.com/datasets/454s2vw255/1) and URLs linking scripts with data are added to each R code and github README.md file.
