# Supplementary material for: Protein-coding genes in humans and model mammals (mouse, rat and pig): gene identifiers and disambiguation of gene nomenclature retrieved from the Ensembl genome browser
Source: BMC Genomics. 2025 Dec 17;27:70. doi: 10.1186/s12864-025-12329-8 (PMC12822150; doi:10.1186/s12864-025-12329-8)
Supplement: Supplementary file 1 — Supplementary Material 1. [file 12864_2025_12329_MOESM1_ESM.docx]

**Supplementary file 1. Supplementary figures for** **“Protein-coding genes in humans and model mammals (mouse, rat and pig):** **gene identifiers and** **disambiguation of gene nomenclature retrieved from the Ensembl genome browser”.**


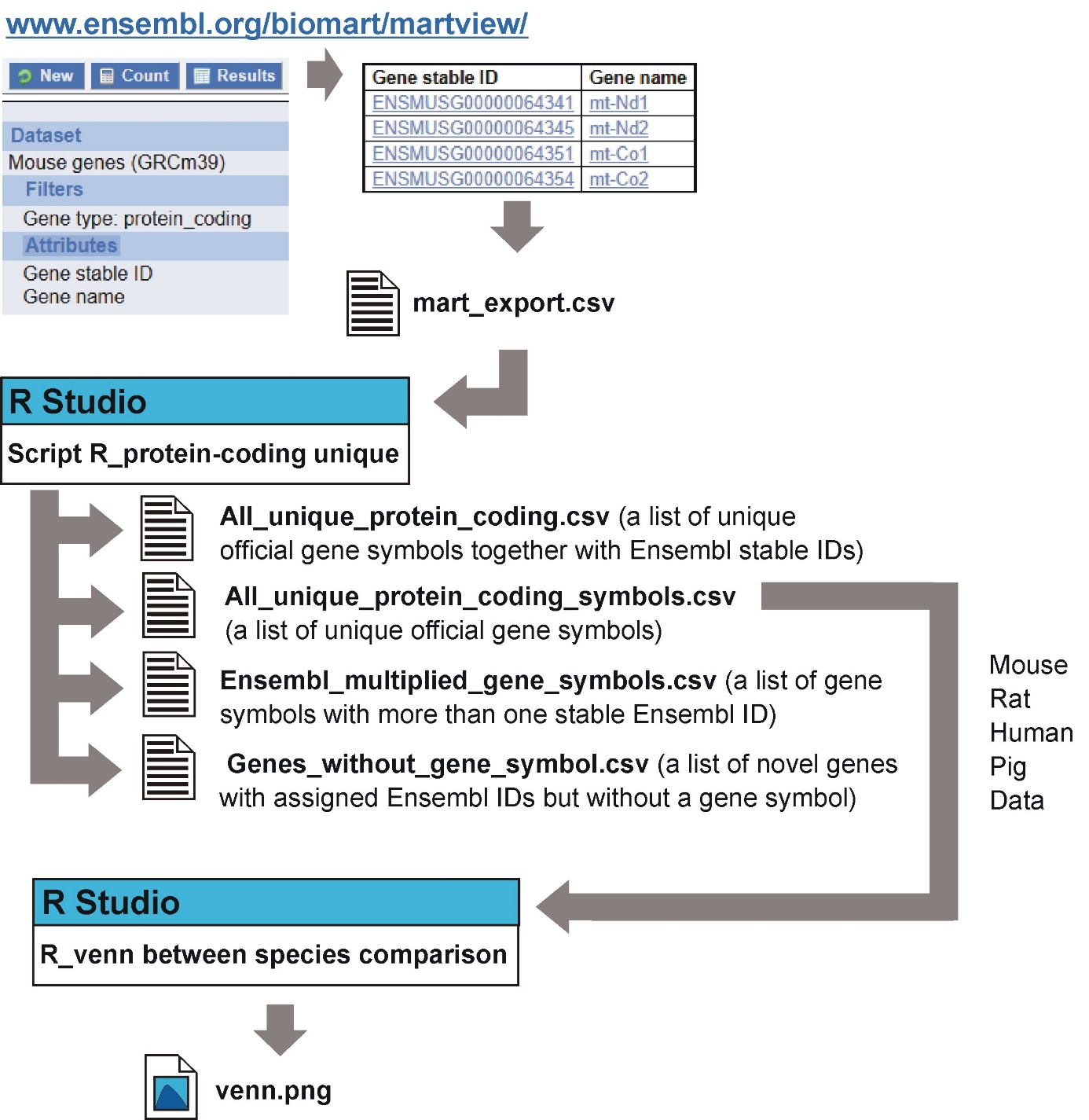


**Supplementary Figure 1. Summary of protein-coding genomes.**


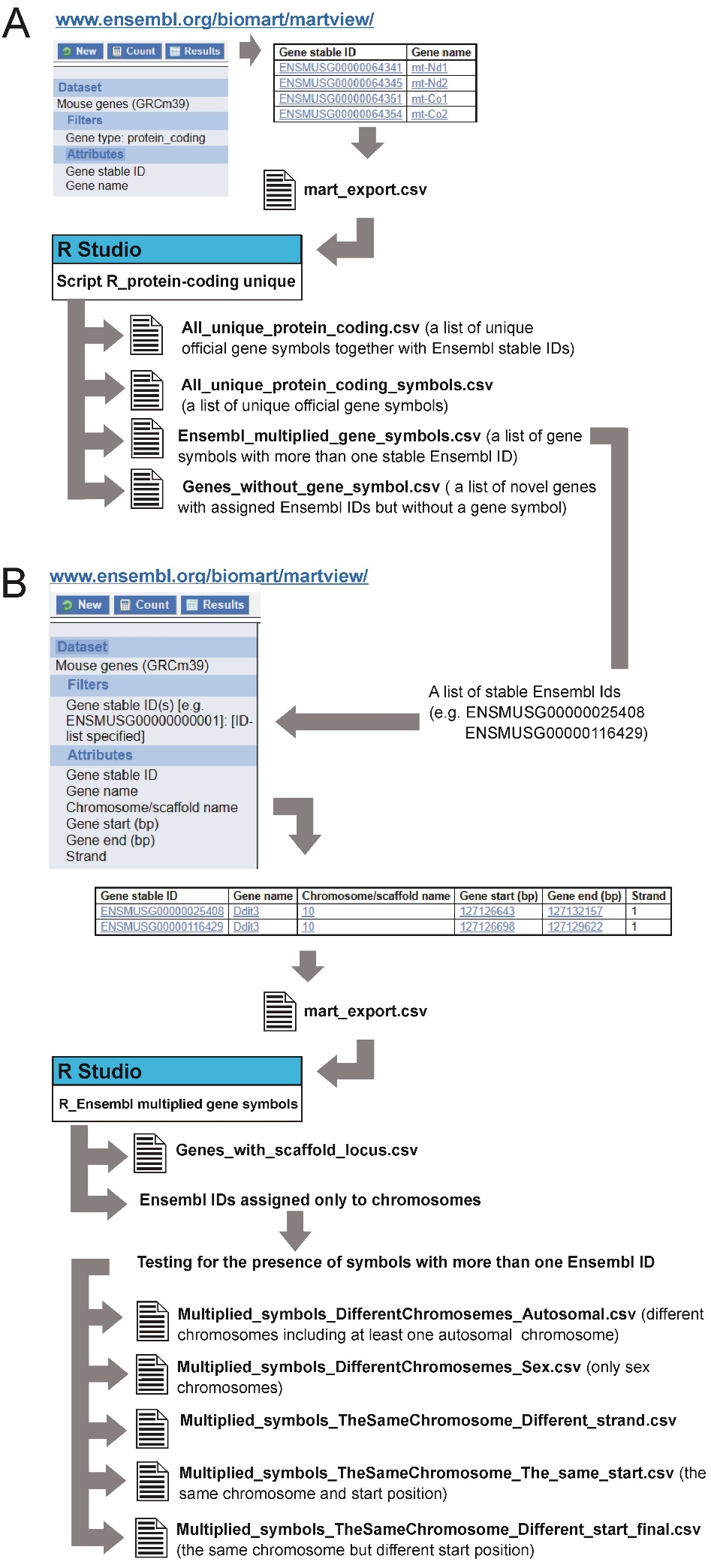


**Supplementary Figure 2. Genomic localization of genes with multiple Ensembl IDs.**


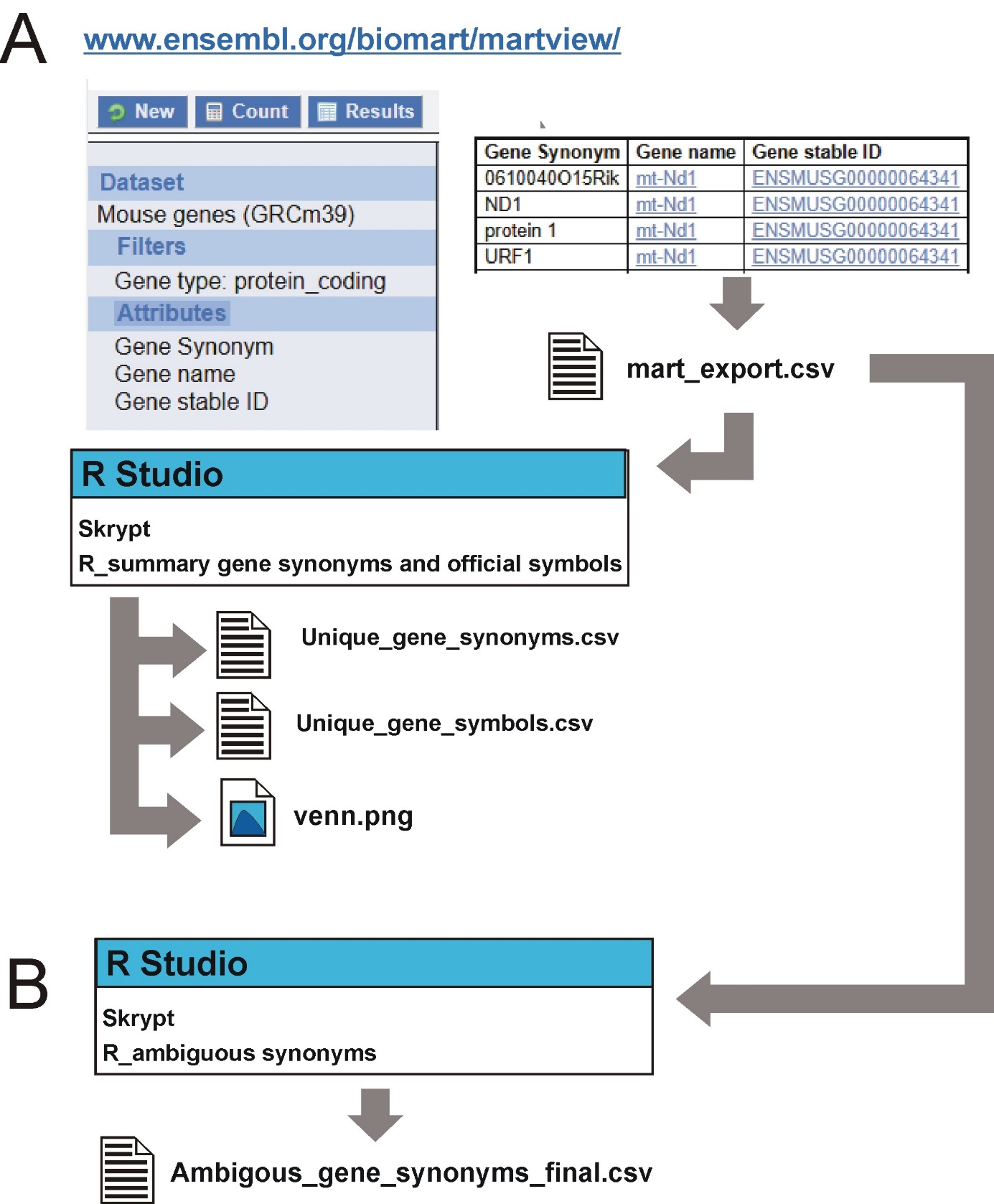


**Supplementary Figure 3. Summary of gene synonyms and official symbols.**


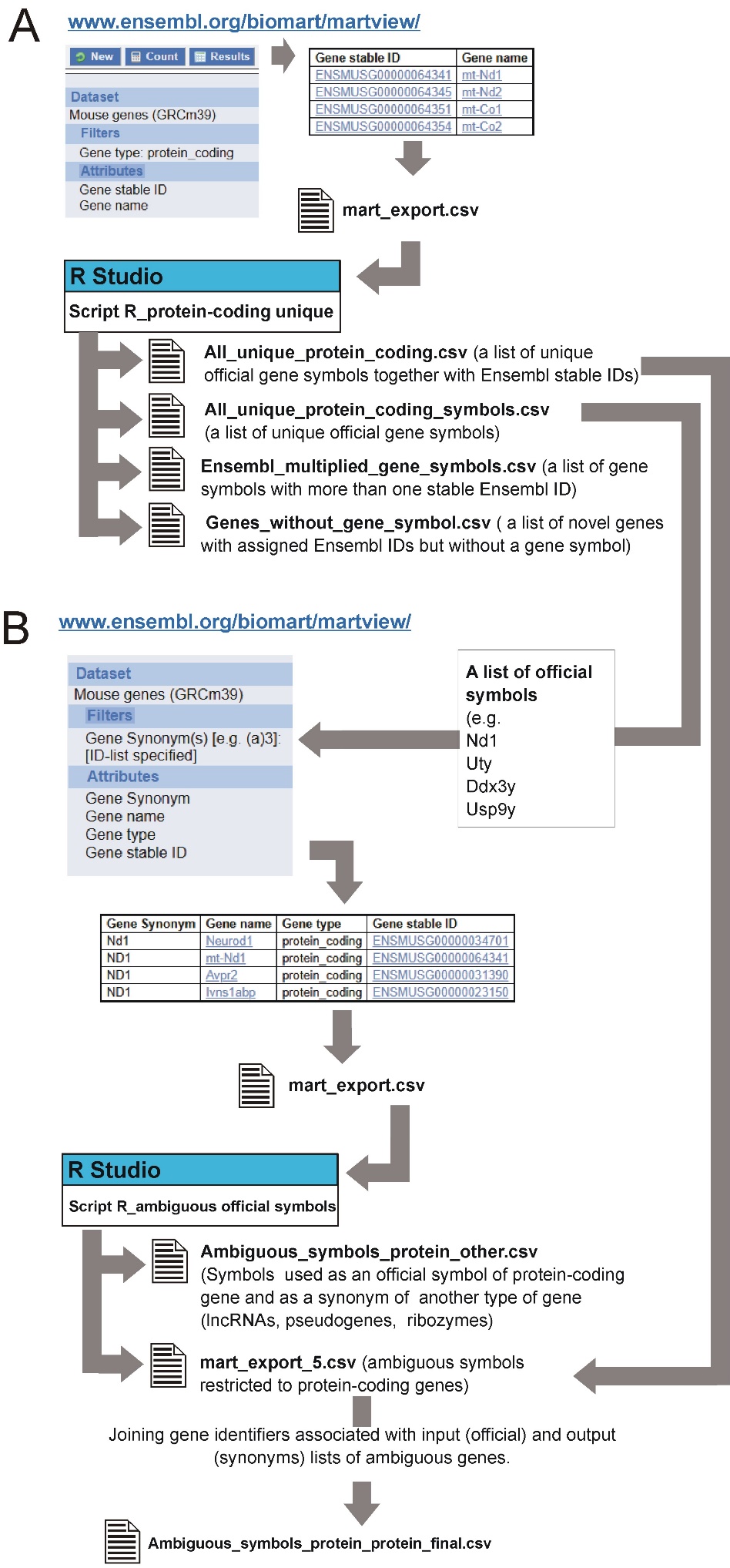


**Supplementary Figure 4. Ambiguous official symbols.**


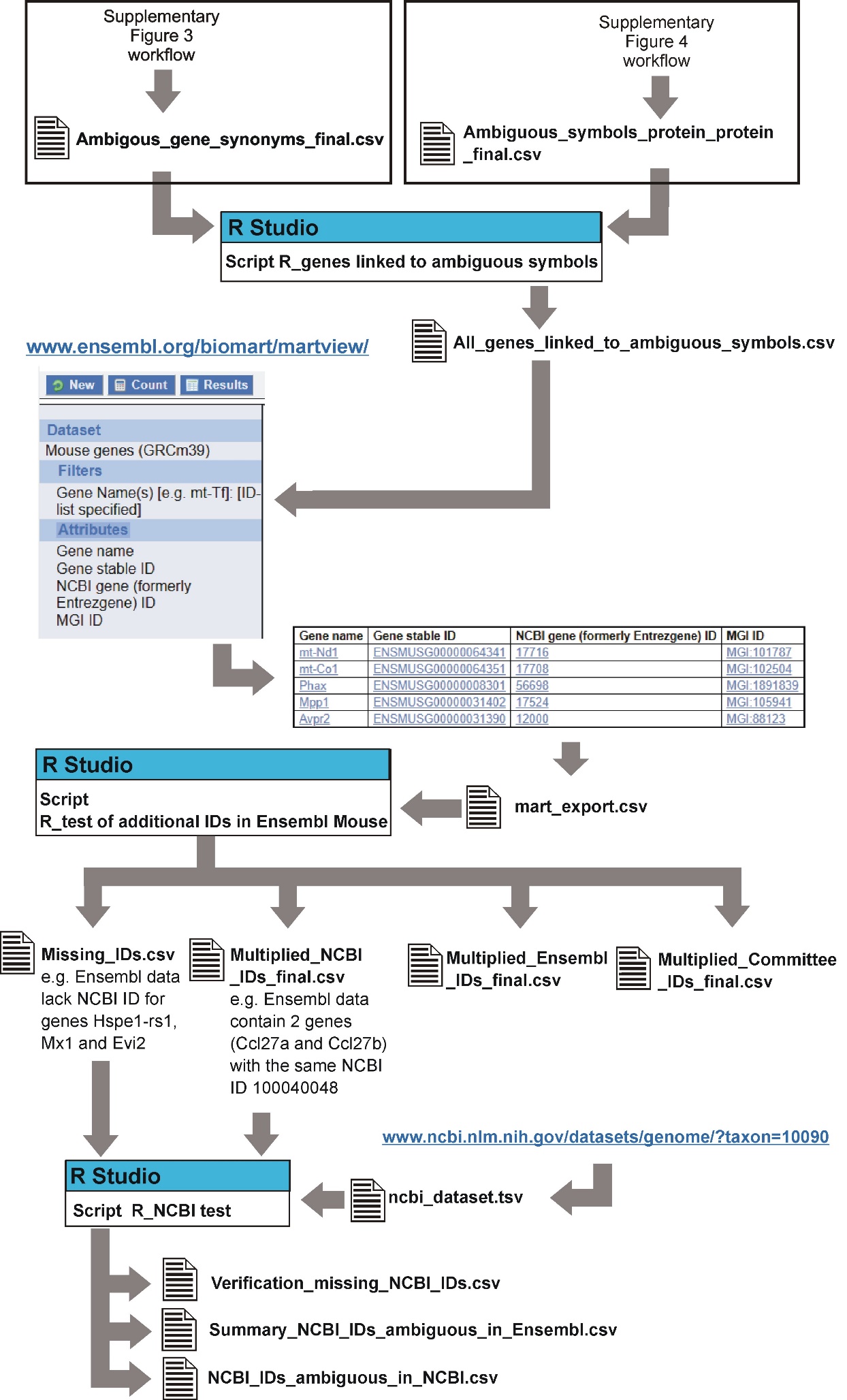


**Supplementary Figure 5. Verification of additional gene IDs.**


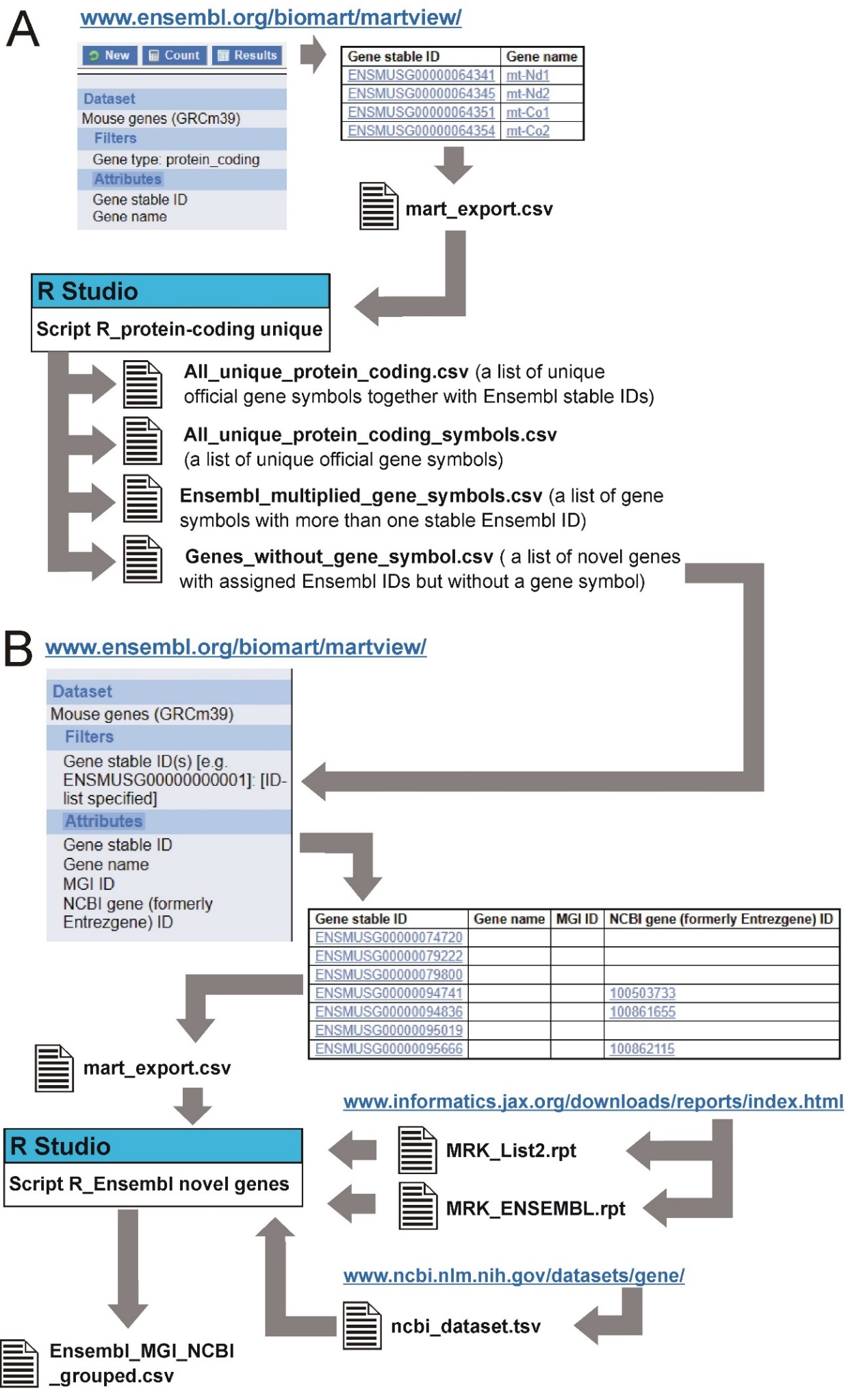


**Supplementary Figure 6. Ensembl genes without a symbol.**
