# Supplementary material for: Protein-coding genes in humans and model mammals (mouse, rat and pig): gene identifiers and disambiguation of gene nomenclature retrieved from the Ensembl genome browser
Source: BMC Genomics. 2025 Dec 17;27:70. doi: 10.1186/s12864-025-12329-8 (PMC12822150; doi:10.1186/s12864-025-12329-8)
Supplement: Supplementary file 2 — Supplementary Material 2. [file 12864_2025_12329_MOESM2_ESM.docx]

**Supplementary file 2. BiomaRt troubleshooting.**

**1. Obsolete software**

biomaRt error information:

Error in `collect()`:

! Failed to collect lazy table.

Caused by error in `db_collect()`:

! Arguments in `...` must be used.

x Problematic argument:

* ..1 = Inf

Solution: Updating R packages with command

library(BiocManager)

install("BiocFileCache")

More information is available at <https://support.bioconductor.org/p/9154901/> and

<https://stackoverflow.com/questions/77370659/error-failed-to-collect-lazy-table-caused-by-error-in-db-collect-using>

In case of further problems It may be necessary to update the biomaRt package.

Additional problems with updating R packages may result from obsolete R software and RStudio and, therefore, may require further software updating.

**2. Malfunctioning of Ensembl servers**

We have encountered several error messages appearing during the work with the biomaRt package. These problems are transient and resolve without any changes to the script. The solution is waiting until the problem is fixed at Ensembl servers. Below are examples of such error messages.

# Example 1

Ensembl site unresponsive, trying asia mirror

Error in 'checkDataset(dataset = dataset, mart = mart)':

The given dataset: rnorvegicus_gene_ensembl , is not valid. Correct dataset names can be obtained with the listDatasets() function.

# Example 2

Error in '.processResults(postRes, mart = mart, hostURLsep = sep, fullXmlQuery = fullXmlQuery, ':

Query ERROR: caught BioMart::Exception::Database: Error during query execution: Table 'mmusculus_gene_ensembl__gene__main' is marked as crashed and should be repaired

# Example 3

Error in '.processResults(postRes, mart = mart, hostURLsep = sep, fullXmlQuery = fullXmlQuery, ':

Query ERROR: caught BioMart::Exception::Database: Error during query execution: Incorrect key file for table 'ensembl_mart_113/hsapiens_gene_ensembl__external_synonym__dm.MYI'; try to repair it

# Example 4

Ensembl site unresponsive, trying asia mirror

Ensembl site unresponsive, trying useast mirror

Error in '.chooseEnsemblMirror(mirror = mirror, http_config = http_config)':

Unable to query any Ensembl site
